# A resurrection of the Haber–Weiss reaction

**DOI:** 10.1038/s41467-021-27823-2

**Published:** 2022-01-19

**Authors:** Willem H. Koppenol

**Affiliations:** 1 Emeritus, Swiss Federal Institute of Technology, Zürich, Switzerland; 2 Present Address: Schwändibergstrasse 25, 8784 Braunwald, Switzerland

**Keywords:** Pollution remediation, Thermodynamics, Pollution remediation

**arising from** Zhao et al. *Nature Communications* 10.1038/s41467-020-20071-w (2020)

As Elimelech et al.^1^ point out, there is a legitimate need for a method that produces singlet dioxygen (^1^Δ_g_O_2_) efficiently, because this species plays a role in a research fields like environmental science and biochemistry. They^1^ describe a flow-through filtration process whereby singlet dioxygen is generated electrochemically. The mechanism proposed by Elimelech et al.^1^ for its production is based on the reduction of hydrogen peroxide by superoxide, the infamous Haber–Weiss reaction, and is therefore incorrect. Furthermore, the evidence for formation of singlet dioxygen is questionable.

The mechanism, shown in Fig. 4 of that publication^1^, starts with reduction of O_2_ at the cathode to O_2_^•−^ and H_2_O_2_. H_2_O_2_ is, of course, also formed by the spontaneous disproportionation of O_2_^•−^. Subsequently, ^1^Δ_g_O_2_ is proposed to be produced via reaction 1:

1 $${{{{{{{\rm{O}}}}}}}_{2}}^{{{\bullet- }}}+{{{{{{\rm{H}}}}}}}^{+}\,{+\,{{{{{\rm{H}}}}}}}_{2}{{{{{{\rm{O}}}}}}}_{2}\,{\to }\,^{1}{\Delta }_{{{{{{\rm{g}}}}}}}{{{{{{\rm{O}}}}}}}_{2}+{{{{{{\rm{HO}}}}}}}^{{{\bullet }}}+{{{{{{\rm{H}}}}}}}_{2}{{{{{\rm{O}}}}}}$$

This reaction became known as the Haber–Weiss reaction (with ^1^Δ_g_O_2_ or^3^Σ_g_^−^O_2_) and was proposed in 1931^2^. It proceeds with a rate constant of at best 1 M^−1^s^−1^ ^3^, and cannot compete^4^ with the rapid and spontaneous^5^ disproportionation of O_2_^•−^.

Elimelech et al. cite my 1976 publication^6^ in support for formation of ^1^Δ_g_O_2_. Indeed, I wrote that reaction (1) is thermodynamically possible. However, the standard Gibbs energies of formation of HO^•^ and O_2_^•−^ have been determined more accurately since then^7^, with the result that reaction 1 with O_2_ in the singlet state is thermodynamically not possible^3,8^.

Elimelech et al. report that insignificant amounts of HO^•^ were detected. This should have led them to reject Reaction 1, because for every ^1^Δ_g_O_2_ also one HO^•^ is formed. Could it be that the terephthalate concentration these authors used to detect HO^•^ was insufficient? If ^1^Δ_g_O_2_ was detected with micromolar concentrations of furfuryl alcohol, then HO^•^ should also have been seen, given that the rate constants of ^1^Δ_g_O_2_ and HO^•^ with furfuryl alcohol and terephthalate are similar, that terephthalate was present in millimolar concentrations, and that unlike HO^•^, ^1^Δ_g_O_2_ is quenched in water at a rate of 2.7 • 10^5^ s^−1^ ^9^. Although irrelevant at this stage, the notion that HO^•^ reacts with terephthalate to yield hydroxyterephthalate is incorrect. Instead, an adduct is formed that needs to be oxidised to yield hydroxyterephthalate.

The authors base their conclusion that ^1^Δ_g_O_2_ is formed also on the reaction of the latter with 2,2,6,6-tetramethylpiperidine, but do not discuss the possibility that this compound may be oxidised at the anode and then yields 2,2,6,6-tetramethyl-4-piperidinol-*N*-oxyl after reaction with O_2_^10^. Formation of any products from ^1^ΔgO_2_ would have been enhanced in D_2_O where ^1^ΔgO_2_ lives much longer, or decreased by addition of a quencher, such as 1,4-diazobicyclo[2.2.2]octane.

Thermodynamically, a simpler route to ^1^Δ_g_O_2_ could be the oxidation of O_2_^•−^ to ^1^Δ_g_O_2_ at the anode, because the electrode potential of the couple ^1^Δ_g_O_2_(aq)/O_2_^•−^ is +0.81 V^11^. In contrast, the spontaneous disproportionation of O_2_^•−^ is not an alternative, as the yield of ^1^Δ_g_O_2_ varies from not detectable to extremely low, as recently reviewed^8^.

If we assume that is ^1^Δ_g_O_2_ formed, we may ask: how much? Given the consumption of furfuryl alcohol, the rate constant of the reaction of this compound with ^1^Δ_g_O_2_, the quenching rate constant for ^1^Δ_g_O_2_ in water^9^, and the flow rate, one arrives at a low nanomolar steady-state concentration of ^1^Δ_g_O_2_. This calculation also shows that only 1.8% of all ^1^Δ_g_O_2_ reacts with furfuryl alcohol.

Experimental conditions and nomenclature need to be discussed too. Elimelech et al^1^. use scavengers at a single concentration, with the exception of terephthalate which was used at two concentrations. These experiments do not prove that ^1^Δ_g_O_2_, O_2_^•−^, H_2_O_2_, and HO^•^ are formed, because such a non-dose-dependent approach only gives an indication. The authors refer to these scavengers as specific for a particular species. Since HO^•^ reacts with nearly everything at high rates, such terminology is inappropriate. Indeed, N_3_^−^ quenches ^1^Δ_g_O_2_, but it also reacts with HO^•^, as do *p*-benzoquinone and catalase. It would have been beneficial if the authors had consulted the Solution Kinetics Database of the National Institute of Standards and Technology^12^ for the relevant rate constants. The description of ^1^ΔgO_2_ as “possessing an empty π* orbital”^1^ is simplistic^13^. When electrofiltration was carried out to remove substances, temperature and pH were not mentioned. The word “quenching” is used as a synonym of “scavenging”, which is incorrect^14^. The term ROS for Reactive Oxygen Species, although widespread, is misleading as neither O_2_^•−^ nor H_2_O_2_ are reactive, as argued before^15^. A study of the thermodynamics and kinetics of reactions of small, short-lived, oxygen-containing species will illustrate this.

In summary, it is not impossible that Elimelech et al. produced singlet dioxygen, but certainly not via the oxidation of O_2_^•−^ by H_2_O_2_.
